# *In Vivo* Volatile Organic Compound Signatures of *Mycobacterium avium subsp*. *paratuberculosis*

**DOI:** 10.1371/journal.pone.0123980

**Published:** 2015-04-27

**Authors:** Andreas Bergmann, Phillip Trefz, Sina Fischer, Klaus Klepik, Gudrun Walter, Markus Steffens, Mario Ziller, Jochen K. Schubert, Petra Reinhold, Heike Köhler, Wolfram Miekisch

**Affiliations:** 1 Department of Anaesthesia and Intensive Care, University Medicine Rostock, Rostock, Germany; 2 Institute of Molecular Pathogenesis, Friedrich-Loeffler-Institut (Federal Research Institute for Animal Health), Jena, Germany; 3 Friedrich-Loeffler-Institut (Federal Research Institute for Animal Health), Greifswald, Germany, and Biomathematics Working Group,Insel Riems, Germany

## Abstract

*Mycobacterium avium* ssp. *paratuberculosis* (MAP) is the causative agent of a chronic enteric disease of ruminants. Available diagnostic tests are complex and slow. *In vitro*, volatile organic compound (VOC) patterns emitted from MAP cultures mirrored bacterial growth and enabled distinction of different strains. This study was intended to determine VOCs *in vivo* in the controlled setting of an animal model. VOCs were pre-concentrated from breath and feces of 42 goats (16 controls and 26 MAP-inoculated animals) by means of needle trap microextraction (breath) and solid phase microextraction (feces) and analyzed by gas chromatography/ mass spectrometry. Analyses were performed 18, 29, 33, 41 and 48 weeks after inoculation. MAP-specific antibodies and MAP-specific interferon-γ-response were determined from blood. Identities of all marker-VOCs were confirmed through analysis of pure reference substances. Based on detection limits in the high pptV and linear ranges of two orders of magnitude more than 100 VOCs could be detected in breath and in headspace over feces. Twenty eight substances differed between inoculated and non-inoculated animals. Although patterns of most prominent substances such as furans, oxygenated substances and hydrocarbons changed in the course of infection, differences between inoculated and non-inoculated animals remained detectable at any time for 16 substances in feces and 3 VOCs in breath. Differences of VOC concentrations over feces reflected presence of MAP bacteria. Differences in VOC profiles from breath were linked to the host response in terms of interferon-γ-response. In a perspective *in vivo* analysis of VOCs may help to overcome limitations of established tests.

## Introduction

*Mycobacterium avium* ssp. *paratuberculosis* (MAP) is the causative agent of paratuberculosis, a chronic enteric disease of ruminants (also called Johne’s disease). In the US, Johne’s disease causes an estimated loss of more than two hundred million dollars per year due to reduced productivity of dairy cattle [1]. Being intestinal pathogens these bacteria are also suspected to contribute to the pathogenesis of Crohn’s disease, a chronic bowel disease in humans [2–5].

The currently established diagnostic methods for paratuberculosis have limitations. Despite state-of-the-art technologies, *in vivo* diagnosis of paratuberculosis demands either fecal shedding of the organisms or sero-conversion, which both occur only irregularly during the clinically non-apparent phase of the disease [6–9]. Cultural detection of MAP in feces or in tissue samples after necropsy is labor intensive and time consuming, while the sensitivity of PCR methods can be affected by low and variable numbers of bacteria in feces and the co-purification of PCR inhibitors during DNA extraction [10]. Novel diagnostic methods, therefore, could gain considerable importance for animal and human health.

More than 300 different VOCs were found in headspace over bacterial cultures [9, 11], feces [12–15] or in breath. Fecal infections with pathogens like *Clostridium difficile* [12, 16, 17], *Campylobacter jejuni* [12, 17], rotavirus, enteric virus [17] and *Vibrio cholera 01* [13] as well as chronic bowel diseases like Crohn’s disease, ulcerative colitis, irritable bowel syndrome [14, 15, 18] were investigated in the past decade. A general problem in analyses performed with samples from feces lies in the fact that the predominant part of gut microbiota are commensal bacteria such as E. coli. As a considerable fraction of VOCs found in feces will, therefore, be generated by these bacteria, a well matched control group has to be analyzed in order to identify VOC patterns of pathogenic bacteria.

VOC patterns in breath have been proposed for identification of tuberculosis, the most important mycobacterial infection in humans [19]. Seven substances were found in breath gas discriminating smear positive from smear negative patients *in vivo*. Two of these substances were also detected in headspace over sputum [20].

In animals, differential ion mobility spectroscopy (DMS) was applied *in vivo* for the identification of MAP infection. In this study, exhaled breath and headspace over feces of goats were analyzed. The composition of VOC patterns differed significantly between chronically infected and non-infected animals [8]. Similar attempts were made with electronic-nose systems (e-nose) analyzing headspace over serum samples of cattle [21].

As unequivocal substance identification is not possible by means of unspecific techniques such as e-nose or DMS, the impact of different substances onto those “VOC features” is still unclear. In addition, the role of potential contaminations from ambient air, previous exposure or medication has to be taken into account. To solve these problems, analytical methods being sensitive and specific enough to quantify and to identify VOCs in trace levels have to be applied. These requirements are optimally met by gas chromatography mass spectrometry (GC-MS) [6, 22, 23].

In recent studies we described VOC patterns consisting of 34 VOCs as potential marker sets for detecting *in vitro* growth of MAP. VOCs were collected in headspace over cultures and analyzed using solid phase microextraction (SPME) in combination with gas chromatography mass spectrometry (GC-MS) [9]. The VOC-patterns mirrored bacterial growth and also enabled distinction of different strains. Transfer of results from *in vitro* tests to *in vivo* may cause crucial misinterpretations if interactions with the host organism or methodological influences such as effects of sample storage or conversion of substances during analysis, are not addressed properly [24, 25]

Therefore, the present study was intended to apply VOC analyses *in vivo* in the controlled setting of an animal model for MAP infection. The following issues were addressed in detail:

- Are there differences between inoculated and non-inoculated animals in terms of VOC profiles in breath and feces?
- Do VOC patterns over feces and in breath correspond to each other?
- Do VOC signatures change during the course of infection?
- Can the previously described VOC markers emitted from MAP cultures be detected in the *in vivo* setting?

## Animals, Materials and Methods

### Animals

All 42 goats belonged to the same domestic race (Thüringer Wald Ziege) and were purchased from a local goat holding. Twenty six goats were inoculated with *Mycobacterium avium* ssp. *paratuberculosis* (strain JII-1961) essentially as described elsewhere [7]. In brief, they received doses of 10 mg bacterial wet mass of the MAP strain ten times every two to three days. Each individual dose was suspended in 50 mL of pre-warmed milk replacer and was administered orally prior to regular morning feeding. The overall bacterial inoculum amounted to 2.6 x 10^8^ cfu per animal. Additionally, 16 goats were not inoculated and were considered as healthy controls. Inoculated and non-inoculated goats were kept in separate stables. The 26 inoculated goats were divided into two groups and kept in different stables. Animal husbandry, preparation of inoculation batches as well as inoculation procedures were carried out as described recently [7]. Control animals were about four weeks younger than the inoculated group of animals.

Twelve, 24 and 36 weeks after the end of inoculation (wpi) seven and twice six inoculated animals and three controls each were dissected. The remaining inoculated (n = 7) and control animals (n = 7) were dissected at the end of the experiment at 52 wpi. Infection was confirmed in 25/26 of the MAP-inoculated goats by cultural isolation of MAP from tissue samples. MAP was not recovered from any of the control animals [7].

#### Ethics statement

This study was carried out in strict accordance with European and National Law for the Care and Use of Animals. The protocol was approved by the Committee on Ethics of Animal Experiments and Protection of Animals of the State of Thuringia, Germany (Permit Number: 04-001/11). All experiments were done in containment of biosafety level 2 under supervision of the authorized institutional Agent for Animal Protection. During the entire study, every effort was made to minimize suffering of the animals.

### Materials

#### Equipment

In order to identify and quantify volatile organic compounds we adapted microextraction techniques for pre-concentration and applied GC-MS for analysis. For VOC determination in the headspace above feces, solid phase microextraction (SPME) was applied. For the breath analyses, needle trap microextraction (NTME) was used.

SPME fiber assemblies (PDMS-Carboxen; 75μm) and SPME Injection sleeves (0.75mm ID, part. no.: 2–6375.05) were bought from Supelco (Bellefonte, USA). A SPME-auto-sampler (Combi-PAL, CTC-analytics, Zwingen, Switzerland) was used.

Needle trap devices (NTDs) packed with 2 cm of a copolymer of methacrylic acid and ethylene glycol dimethacrylate were obtained from Shinwa Ltd., Japan (NeedleEx) [23]. A custom-made NTD-heating-station, NTD-auto-sampler, Teflon-caps and magnetic cap with a Teflon-inlet for sealing of NTDs were bought from PAS Technology (Magdala, Germany). The 20-mL-headspace-vials and Teflon-coated rubber septa in combination with magnetic crimp caps were purchased from Gerstel GmbH & Co.KG (Muelheim/Ruhr, Germany).

Gas chromatographs (GC) (model-no.: 7890A) in combination with inert XL mass selective detectors (MS) (model-no.: 5975C), long life non-stick septa and non-stick Liner O-rings were bought from Agilent Technologies (Boeblingen, Germany).

A CP-Pora Bond Q Fused Silica Column (25 m, 0.32 mm, Varian) was applied for the analysis of the SPME samples. A RTX-624 (60 m; 0.32 mm; 1.8 μm film thickness) Restek, Bad Soden, Germany) capillary column was used for NTME analysis.

#### Reference substances

Identities of all VOCs considered as potential marker substances were confirmed through analysis of pure reference substances.

Acetone, 2-butanone, hexanal, nonanal, isoprene and benzene were acquired from Ionimed Analytik GmbH (Innsbruck, Austria). Butane, pentane and hexane were bought from Supelco (Bellefonte, USA). Methyl-isobutyl-ketone, 1-propanol, styrene, 3-octanone, 2-heptanone, furan, heptane, methylacetate, 2-methylfuran, 2-ethylfuran, 2-pentylfuran, 2-pentanone, 2-hexanone, 3-hexanone, 3-methyl-2-butanone, 3-methyl-2-pentanone, 2-propanethiol and dimethylsulfide were purchased from Fluka/Sigma-Aldrich (Steinheim, Germany). 2-Methyl-butanal and dimethyldisulfide were bought from Abbott GmbH & Co.KG (Wiesbaden, Germany). Ethylbenzene was acquired from Merck (Darmstadt, Germany). 3-methylfuran and 3-methylbutanal was purchased from TCI Europe N.V. (Zwijndrecht, Belgium).

### Methods

#### Sampling

Five measuring campaigns in the infected animals and the control group were carried out during a period of 8 months. Table 1 shows a detailed overview of breath gas and feces sampling. Sampling from inoculated goats and healthy animals was always done at the same day, irrespective of the individual age of the goats.

**Table 1:** Overview of breath gas samples (b) and feces samples (f) taken from goats.

weeks after inoculation		18	29	33	41	48	Σ
		sampled animals					
**inoculated animals**	**b**	11	6	13	7	6	**43**
	**f**	19	13	13	7	5	**57**
**non-inoculated animals**	**b**	13	8	10	7	7	**45**
	**f**	16	10	10	7	7	**50**

#### Feces

Samples from headspace over feces were pre-concentrated by means of SPME and analyzed by means of GC-MS. Feces were collected into 20 mL headspace vials and sealed with Teflon-coated rubber septa in combination with magnetic crimp caps. Carboxen/PDMS-containing SPME-fibers were used for all measurements. Before SPME-fibers were used for the first time, they were conditioned in a GC-injector at 300°C for 1h. Afterwards fibers were retracted and tips of the fibers were sealed until usage. Feces samples were conditioned at 42°C for 3 min. Fibers were pierced through the septum and exposed to the headspace of the feces for 7 min. The sample was agitated during equilibration with 500 rpm using the CTC-PAL system. Afterwards fibers were retracted and immediately injected into the hot injector for measurement. Before each reuse, SPME-fibers were reconditioned again at 290°C in the GC injector for 30min.

Blank runs of the fibers were performed on every day before the measurements to ensure that the SPME-coating was clean and that no uncontrolled bleeding of column, septa or fiber took place. In order to control the quality of SPME-fibers, a consistent concentration (ppb-range) of 2,3-dimethyl-1,3-butadiene in methanol was analyzed at the beginning and at the end of every GC-MS sampling queue.

#### Breath gas

**Fig 1:**
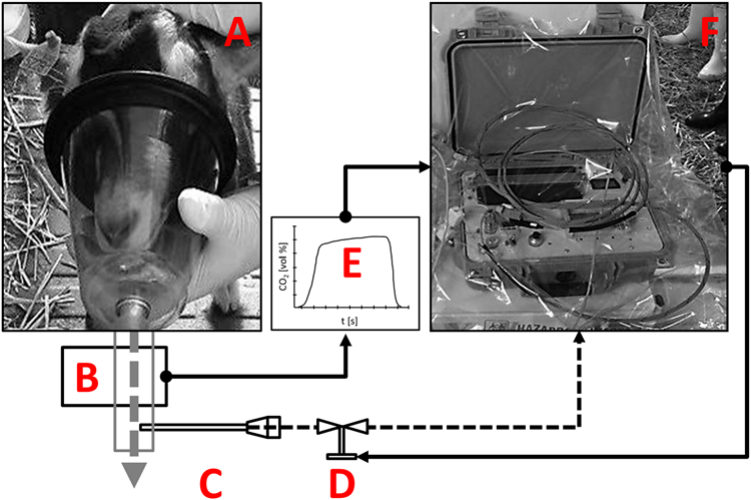
Experimental set up for alveolar breath sampling in goats. A—sampling mask; B—CO_2_-sensor; C—Needle Trap Device (NTD); D—CO_2_ triggered flow valve; E—Capnogard for time resolved CO_2_-monitoring; F—sampling box; dashed arrows represent air flows; continuous arrows represent electronic signals.

An automated alveolar sampling device (PAS Technology, Magdala) [26] was used for alveolar NTD breath sampling (Fig 1).

Sixty mL of alveolar gas were collected onto the NTD by means of the automated CO_2_ controlled device. The device was connected to a fast responding mainstream capnometer (Philips Respironics, response time < 60 ms). In the alveolar phase of exhalation a valve was opened and breath gas was drawn through the NTD. Sample volume was controlled by means of a mass flow controller. The flow rate during sampling was 20 mL/min. After sampling the NTDs were sealed at the luer lock end by means of magnetic caps with Teflon-inlets. On the tip end, NTDs were sealed with Teflon-caps. The same procedure of breath sampling without CO_2_ controlled opening of the valve D (Fig 1) was used for generation of at least two room air samples per day and per stable.

Prior to first usage NTDs were conditioned in a heating device (PAS technology) for at least 20 hours at 195°C under permanent helium flow (1 bar front pressure). After conditioning, NTDs were sealed at both ends with Teflon-caps. Before actual use NTDs were reconditioned for 30 minutes at 195°C.

#### SPME-GC-MS analysis of feces

GC-MS (Agilent MSD) was used for separation and detection of VOCs desorbed from SPME-fibers. SPME fibers were thermally desorbed at 290°C in the GC-injector. During sample injection the injector was operated in splitless mode for 60s at a head pressure of 0.643 bar, resulting in a column flow rate of 1.7 mL/min. After the injection phase a split ratio of 30 was applied for the remaining analysis time. The column temperature program consisted of the following steps: 90°C for 6 min, 15°C/min to 120°C for 1 min, 10°C/min to 140°C for 7 min, 15°C/min to 260°C for 6 min. The mass spectrometer was operated in electron impact ionization (70 eV) mode and recording was done in full scan mode. The mass range was 35–300 amu and the scan rate was 2.73 scans per second. The temperature of the ion source was set to 230°C and the temperature of the transfer line was set to 280°C. Extracted ion counts (EIC) were used to calculate peak areas for all compounds. Peak areas were calculated using Agilent MSD Chemstation (E.02.00.493) Software.

#### NTD-GC-MS analysis of breath gas

GC-MS (Agilent MSD) was used for separation and detection of VOCs desorbed from the NTDs. Teflon-caps at the tip end were stripped off by the NTD-auto-sampler (Concept, PAS Technology, Germany) prior to injection. Automatic desorption of NTDs was realized by automated insertion of NTDs into the injection port [27]. NTDs were thermally desorbed using the expansive flow technique in the injection port of the GC (splitless mode). The temperature of the injector was 200°C [28]. During desorption the injector was operated in splitless mode for 30s at a head pressure of 0.603 bar, resulting in a column flow rate of 1.5 ml/min. After the injection phase a split ratio of 33 was applied for the remaining analysis time. The column temperature program consisted of the following steps: 40°C for 5 min, 8°C/min to 120°C for 2 min, 10°C/min to 220°C, 20°C/min to 250°C for 4 min. The mass spectrometer was operated in electron impact ionization (70 eV) mode and recording was done in full scan mode. Mass range was 50–300 amu and scan rate was 2.73 scans per second. Temperature of the ion source was 250°C and temperature of the transfer line was 240°C. Extracted ion counts (EIC) were used to calculate peak areas for all compounds. Peak areas were calculated using Agilent MSD Chemstation (E.02.00.493) Software.

#### Identification and quantification of marker substances by means of GC-MS

Tentative substance identification was done by means of mass spectral library (NIST 2005 Gatesburg, PA, USA) search. Compounds with concentration differences between inoculated and non-inoculated animals were considered as potential marker substances.

GC retention times and mass spectra of all potential marker compounds were verified through analysis of pure reference substances. The substance lists are given in Table 2. Substances marked with an asterisk (*) in Table 2b had been identified as potential biomarkers for MAP bacteria in a former *in vitro* study [9].

**Table 2:** Retention times, regression coefficients, detection limits and concentration ranges of selected VOC marker substances from breath (a) and feces (b).

substance					VOC-concentration [ppbV]					
	retention time	R^2^	LOD	LOQ	inoculated animals			non-inoculated animals		
					43 measurements			45 measurements		
a) breath	[min]	[/]	[ppbV]		25%	median	75%	25%	median	75%
**1-Propanol**	9.96	0.976	1.41	3.48	184	258	445	69	214	528
**2-Butanone**	10.95	0.993	0.19	0.36	0.39	0.54	0.68	0.48	0.67	0.95
**Acetone**	7.53	0.854	0.25	0.45	22.2	30	38.7	26.7	32.7	38.3
**Benzene**	12.44	0.994	0.50	1.06	†	0.52	0.64	†	0.58	0.75
**Butanal, 2-methyl-**	12.99	0.977	0.08	0.11	†	†	0.24	†	0.33	0.57
**Ethylbenzene**	19.04	0.984	0.11	0.24	†	†	0.13	†	†	0.17
**Hexanal**	17.5	0.995	0.11	0.28	0.45	0.62	0.91	0.49	0.62	0.81
**Nonanal**	25.32	0.954	0.40	0.91	1.44	2.02	3.09	1.51	2.27	3.12
**Styrene**	20.18	0.982	0.02	0.03	0.05	0.09	0.24	0.03	0.06	0.21
**b) feces**					**57 measurements**			**50 measurements**		
Pentane*	13.22	0.999	2.41	11.1	13.5	18.1	23.1	11.8	16.6	21.1
Hexane*	20.58	0.999	4.41	16.3	1.9	10	12.4	†	†	9.4
Heptane*	23.83	0.998	2.64	8.81	7.4	8.9	12.1	6	8.2	10
Acetone*	10.11	0.999	7.06	24.8	613	857	1180	273	379	476
**2-Butanone**	16.06	1.000	0.43	1.26	53	100	169	46	78	107
**2-Pentanone**	22.13	0.998	1.69	5.64	278	416	586	192	276	339
**2-Hexanone**	24.97	0.997	5.39	18.0	117	174	224	59	95	120
2-Heptanone*	26.46	0.999	2.98	9.94	27.4	36.9	46.3	21.5	26	34.2
3-Octanone*	28.36	0.999	22.6	75.5	41.1	58.3	90.6	25.3	51	86.1
**2-Butanone, 3-methyl-**	21.53	0.997	0.53	1.77	23.8	28.5	36.4	29.1	39.4	72.7
**2-Pentatone, 3-methyl-**	24.22	0.997	1.79	5.95	24.1	34.1	63.9	13.4	17.9	27.5
Methyl Isobutyl Ketone*	24.00	0.998	1.26	4.19	9.7	13.2	16.9	6	7.8	10.9
Isoprene*	12.63	1.000	1.22	4.65	20	27.2	35.1	4.2	12.5	22.1
Acetic acid, methyl ester*	11.57	0.999	0.29	0.97	42.5	49.6	61.4	45.6	57.1	72.3
**Sulfide, dimethyl**	10.91	0.997	0.99	3.29	473	869	1316	99	183	360
Disulfide, dimethyl*	22.61	0.993	0.08	0.26	0.5	2	6.5	†	0.4	1.1
Furan*	9.81	0.999	0.10	0.35	1.5	1.8	2.3	1.6	1.9	2.8
Furan, 2-ethyl-*	21.95	0.998	0.19	0.65	8.3	10.1	12.4	4.6	6.1	7.7
Furan, 2-methyl-*	15.82	0.993	9.6	31.8	16.4	20.3	27.6	11.9	14.9	17.2
Furan, 3-methyl-*	16.62	0.996	0.13	0.42	24.1	31.8	40.1	17.3	21	25.6
Furan, 2-pentyl-*	28.40	0.995	4.64	15.5	23.6	28.2	34.5	19.3	24.6	33.2

R^2^—coefficient of determination;LOD—limit of detection;LOQ—limit of quantification

* potential marker substances from a former in vitro study [9]

^†^ values lower than LOD

For quantification, gas standards were prepared in concentrations between 1 and 50 ppbV. For acetone, 3-octanone, 1-propanol, 2-butanone, 2-pentanone, 2-hexanone, 3-methyl-2-pentanone, methylacetate and dimethylsulfide higher concentrations were prepared additionally for calibration. At least five different concentration levels were prepared for calibration of each substance. Calibration mixtures of pure reference materials not available as gaseous standards were prepared by transferring liquid reference substances into an evacuated 100 mL gas bulb by means of a 10μL syringe. The gas bulb was then equilibrated with pure nitrogen. 50 μL of the gas mixture were transferred into a Tedlar-bag filled with 1 L of nitrogen. Different concentration levels were prepared by further dilution with nitrogen. For SPME calibration, 15 mL of these gas standards were transferred into evacuated 20 mL headspace vials and pressure was equilibrated with nitrogen. All gas standards were pre-concentrated by means of SPME under the same conditions as the headspace of the feces samples. For NTD calibration 50 mL of gas standard was pre-concentrated on the NTD device similar to sampling of breath gas.

Limits of detection (LODs) and limits of quantitation (LOQs) for SPME-GC-MS and NTME-GC-MS methods were determined by means of the signal to noise ratio. Noise level was determined experimentally from blank samples. LOD was defined as S/N of 3, LOQ as S/N of 10.

#### Blood samples

Blood samples were collected from inoculated and control animals in regular intervals. 20 mL of heparinized blood were collected from each animal in intervals of four weeks until the end of the experiment starting at 4 weeks after inoculation. The serum antibody response was measured with the ID Screen Paratuberculosis Indirect ELISA (ID Vet, Montpellier, France) according to the instructions of the manufacturer. The antibody response was demonstrated by the sample-to-positive ratio (S/P %). The interferon-γ response after 24 h stimulation of peripheral blood mononuclear cells (PBMC) with Johnin purified protein derivative (JPPD, 4 μg/mL), was measured with an in-house ELISA based on monoclonal capture and detection antibodies against bovine IFN-γ (AbD Serotec, Kidlington, UK).

**Fig 2:**
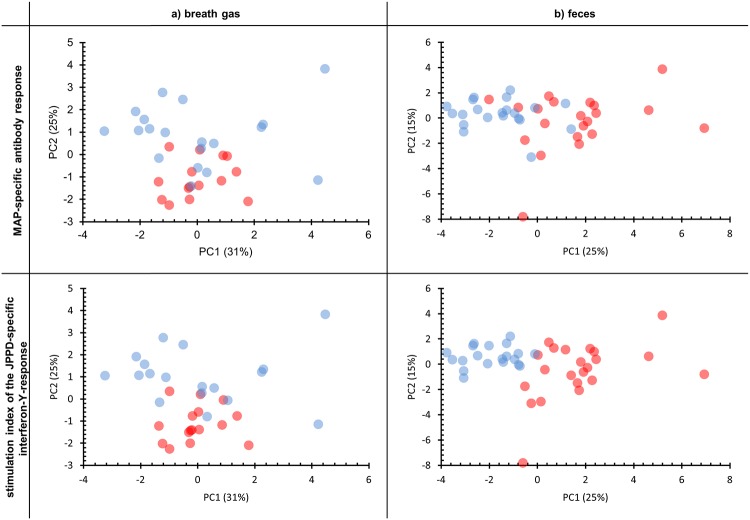
PCA-scatterplots of VOC patterns in breath gas (a) and headspace over feces (b) in relation to MAP-specific antibody levels and to the interferon-γ response. Red dots represent S/P-ratios of the antibody-ELISA higher than 51 and stimulation-indices of the interferon-γ response higher than 1.157, respectively. The loading plot referring to the PCA results from breath gas is given in S1 Fig and the loading plot referring to the PCA analysis of results from feces is given in S2 Fig, respectively. Blue dots represent S/P-ratios of the antibody-ELISA lower than 51 and stimulation-indices of the interferon-γ response lower than 1.157, respectively.

It was assumed that the non-inoculated control group represented a population whose physiological responses were comparable to natural non-infected animals. Inoculated animals were possibly representing progress of infection. Therefore, cut-off-values with high specificity (>99%), estimated via Receiver-operating-characteristics- (= ROC)-analysis, were chosen for both MAP-specific features. These cut-off-values were used for labeling of results in PCA-scatterplots of VOC-measurements which are given in Fig 2.

#### Statistical methods and visualizations

For all statistical methods and visualizations only quantified data were used. Visualization of VOC patterns in two-color heat maps was done with substance concentrations normalized by means of the maximum in the data set.

Principle component analyses (PCA) were performed by means of The Unscrambler 9.7 and 10.3 (Camo Software AS). For that purpose, data were normalized onto standard deviations and cross-validation was applied as validation method.

Mann-Whitney-U-tests, t-tests and ROC-analyses were realized by means of Sigmastat 3.5 as well as SigmaPlot (Version 10.0.0.54; Systat, San Jose, USA). Significance levels were set to α = 0.05. Values of p ≥ 0.05 meant that there were no significant differences between the two tested groups. No significance test was performed if percentiles of regarded groups were lower than LOD. Confidence level for ROC-analysis was set to 99%. In case of normal distribution, t-tests instead of U-tests were performed.

## Results

**Fig 3:**
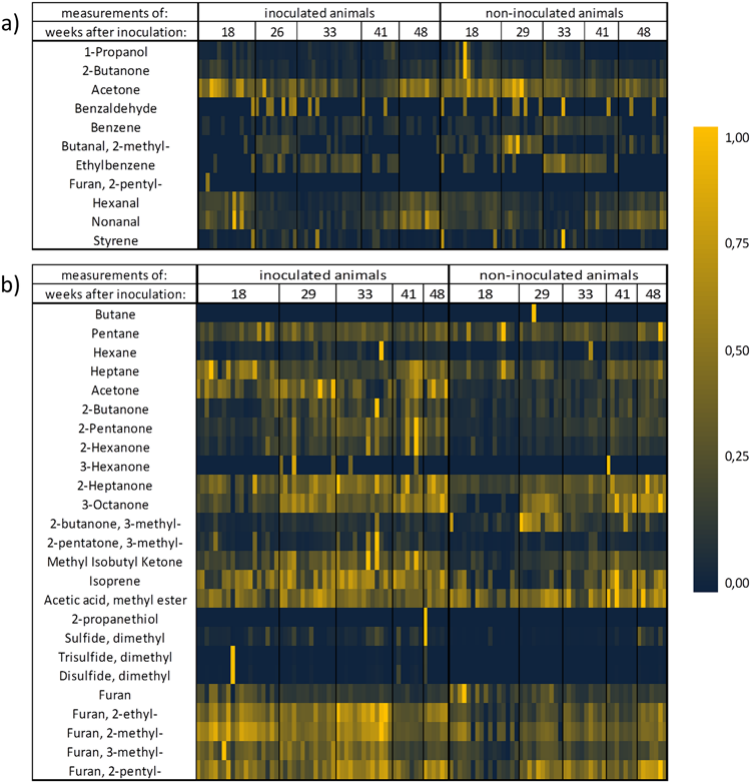
Heat-map of selected VOCs from breath (a) and feces (b) normalized onto maximum of concentrations.

More than 100 substances could be detected in breath and in feces of the animals by means of GC-MS-analysis. Detection limits in the high pptV- or low ppbV- and linear ranges of two orders of magnitude could be achieved. Twenty eight substances could be identified having different concentrations in inoculated and non-inoculated animals. VOC marker patterns changed during infection. Fig 3 shows the normalized VOC concentration patterns of all analyzed samples from headspace over feces and from exhaled breath. Although patterns of most prominent substances such as furans, oxygenated substances and hydrocarbons changed in the course of infection, differences between inoculated and non-inoculated animals remained detectable at any time for 16 substances in feces and 3 VOCs in breath. Table 2 shows the selected marker substances together with the detection/quantitation limits of the method and the measured concentration ranges.

### VOC Patterns in Headspace over Feces

Within the group of 25 substances having different concentrations in the inoculated and non-inoculated groups of animals (Fig 3b) butane, 3-hexanone, 2-propanethiol and dimethyltrisulfide were only detected in a few measurements. These substances were, therefore, not introduced into the final marker set.

Twenty one substances were identified as potential marker substances in headspace over feces (see also Table 2b). Mann-Whitney-U-tests revealed significant differences in substance concentrations between the inoculated and non-inoculated groups of animals (Table 3).

**Table 3:** Mann-Whitney-U-tests for analyses from headspace over feces.

Substances:	p-values	Substances:	p-values
**Pentane**	0.176	**Methyl Isobutyl Ketone**	<0.001
**Hexane**	0.003	**Isoprene**	<0.001*
**Heptane**	0.022	**Acetic acid, methyl ester**	0.898
**Acetone**	<0.001	**Sulfide, dimethyl**	<0.001
**2-Butanone**	0.016	**Disulfide, dimethyl**	<0.001
**2-pentanone**	<0.001	**Furan**	0.275
**2-hexanone**	<0.001	**Furan, 2-ethyl-**	<0.001
**2-heptanone**	<0.001	**Furan, 2-methyl-**	<0.001*
**3-Octanone**	0.161	**Furan, 3-methyl-**	<0.001
**2-Butanone, 3-methyl-**	<0.001	**Furan, 2-pentyl-**	0.057*
**2-Pentatone, 3-methyl-**	<0.001		

*—p-value from t-test

Concentrations of hexane, heptane, acetone, 2-butanone, 2-pentanone, 2-hexanone, 2-heptanone, 3-methyl-2-pentanone, methyl isobutyl ketone, isoprene, dimethyl sulfide, dimethyl disulfide, 2-ethyl-furan, 2-methyl-furan and 3-methyl-furan were significantly higher in headspace over feces of the inoculated group. Significantly lower concentrations for 3-methyl-2 butanone were found in headspace over feces of the inoculated group.

**Fig 4:**
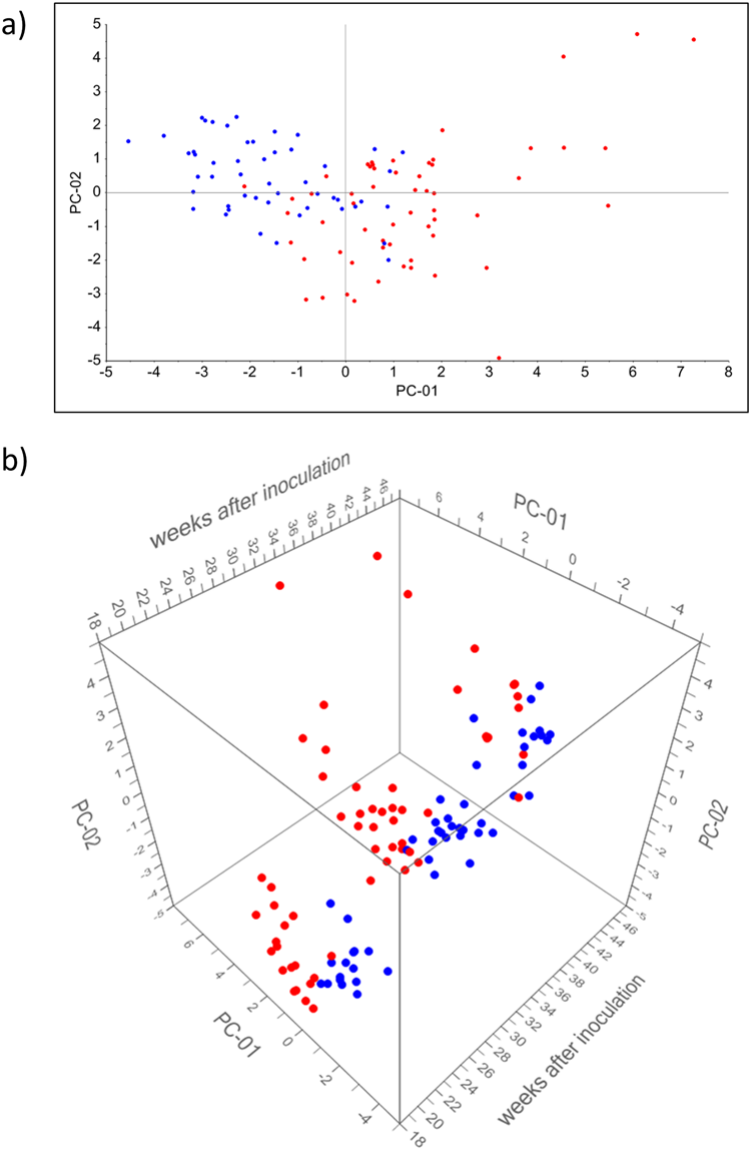
PCA-scatterplots based on VOC-analysis of headspace over feces. PCA (a) was done for substances (Table 3) having significantly different (p < 0.05) concentrations in inoculated and non-inoculated animals. The 3D-scatterplot (b) is derived from the same PCA with respect to weeks after inoculation on the third axis. The loading plot referring to this PCA analysis is given in S3 Fig. Blue dots represent the non-inoculated group. Red dots represent the inoculated group. PC-01 explains 31% and PC-02 explains 16% of variance.

Fig 4 shows scatterplots of the first two principal components of a PCA analysis of the marker sets for inoculated and non-inoculated animals. As shown in Fig 4a discrimination between inoculated and non-inoculated animals is mainly attributed to PC1. The time passed after inoculation had an effect on VOC- patterns (Fig 4b) but did not interfere with the discrimination of inoculated and non-inoculated animals.

### VOC Patterns in Breath Gas

Within the group of eleven substances having different concentrations in the inoculated and non-inoculated groups of animals (Fig 3a) benzaldehyde and 2-pentyl-furan were only detected in a few measurements. Those substances were, therefore, not introduced into the final marker set.

Nine substances were identified as potential marker substances through NTME-GC/MS analysis of breath gas (see also Table 2a). Mann-Whitney-U-tests revealed significant concentration differences (p < 0.05) of three substances between the inoculated and non-inoculated group of animals (Table 4). 2-butanone, benzene and 2-methyl-butanal were higher concentrated in breath gas of the non-inoculated group than in exhaled breath of the inoculated group.

**Table 4:** Mann-Whitney-U-tests for breath gas-measurements.

Substances	p-values
**1-Propanol**	0.458
**2-Butanone**	0.003
**Acetone**	0.064
**Benzene**	0.023
**Butanal, 2-methyl-**	0.002
**Ethylbenzene**	0.32
**Hexanal**	0.372
**Nonanal**	0.881
**Styrene**	0.649

**Fig 5:**
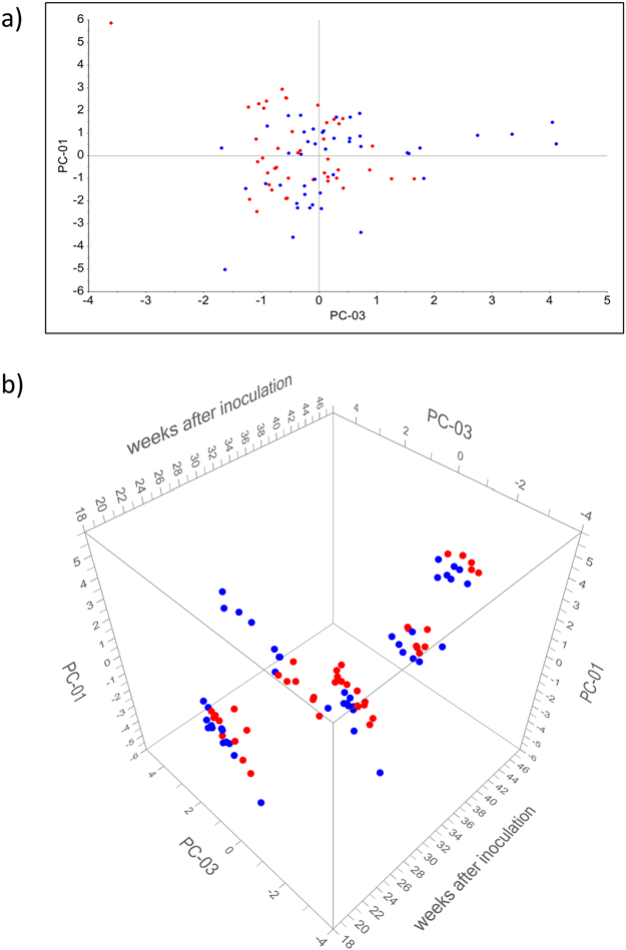
PCA-scatterplots based on VOC-analysis of breath. PCA (a) was done for all substances (Table 2) having different concentrations in inoculated and non-inoculated animals. The 3D-scatterplot (b) is derived from the same PCA with respect to weeks after inoculation on the third axes. The loading plot referring to this PCA analysis is given in S4 Fig. Blue dots represent the non-inoculated group. Red dots represent the inoculated group. PC-01 explains 31% and PC-03 explains 15% of variance.

Fig 5 shows scatterplots of a PCA including all identified substances. No discrimination related to PC 1 and PC 3 was found between inoculated and non-inoculated animals (Fig 5a). As seen in Fig 5b the time after inoculation has more effect on PC1 and PC3 than the bacterial status of animals.

**Fig 6:**
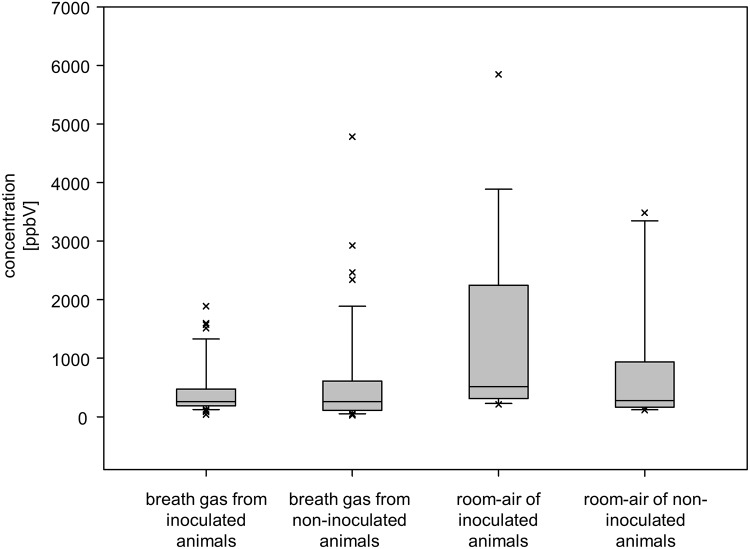
Concentration of 1-propanol in breath gas- and room air-samples.

Additionally, room air of each stable was sampled for estimation of background contaminations. Fig 6 representatively shows concentrations of 1-propanol in the breath of animals and in the air of the corresponding stables.

### Correlation of VOC Patterns with Blood Based Immunological Tests

Fig 2a shows scatterplots of a PCA analysis of breath gas VOC profiles in relation to MAP-specific antibodies and interferon-γ response. PC1 explained 31% and PC2 25% of the variance within the data set. According to the loading plot substances such as nonanal, 2-methyl-butanal, ethylbenzene and benzene had the highest impact on PC1. Styrene, benzaldehyde, 1-propanol and 2-butanone showed the highest loadings on PC2. S/P-ratios of antibody titer and stimulation index of interferon-γ response were positively correlated to PC 2. No correlation was found to PC 1. Table 5 exhibits that concentrations of 2-butanone, acetone and benzene were significantly higher in animals with lower antibody titers and interferon-γ response. Nonanal concentrations were also higher in animals with lower interferon-γ response.

**Table 5:** VOC-concentration ranges and differences in relation to MAP-specific antibody levels and interferon-γ response after stimulation with JPPD.

a) breath	antibody titer (S/P%)							stimulation-index of the JPPD-specific interferon-γ-response						
	≥ 51.02			< 51.02				≥ 1.157			< 1.157			
measurements	15			19				16			18			
percentiles	25%	median	75%	25%	median	75%	p	25%	median	75%	25%	median	75%	p
**1-Propanol**	182.08	202.26	305.02	221.18	325.37	647.68	**0.048**	181.29	199.16	279.77	246.43	417.71	669.23	**0.01**
**2-Butanone**	0.34	0.40	0.53	0.61	0.75	1.08	**<0.001**	0.33	0.41	0.54	0.69	0.76	1.12	**<0.001**
**Acetone**	18.89	24.11	32.66	28.76	32.70	43.21	**0.011**	18.53	23.32	30.22	32.11	34.34	46.93	**<0.001**
**Benzene**	†	0.55	0.61	0.59	0.70	0.82	**0.012**	†	0.55	0.64	0.59	0.71	0.93	**0.009**
**Butanal. 2-methyl-**	†	0.32	0.45	†	†	0.85	**0.776**	†	0.35	0.50	†	†	0.68	**0.83**
**Ethylbenzene**	†	0.12	0.15	†	0.14	0.19	**0.211**	†	0.11	0.15	†	0.16	0.20	**0.084**
**Hexanal**	0.35	0.41	0.51	0.43	0.50	0.74	**0.055**	0.36	0.45	0.51	0.37	0.50	0.77	**0.128**
**Nonanal**	1.32	1.46	1.71	1.26	2.05	2.50	**0.053**	1.30	1.45	1.82	1.29	2.07	2.59	**0.047**
**Styrene**	0.06	0.16	0.49	0.05	0.15	0.63	**0.839**	0.08	0.19	0.75	0.04	0.10	0.39	**0.183**
**b) feces**	**antibody titer (S/P%)**							**stimulation-index of the JPPD-specific interferon-γ-response**						
	**≥ 51.02**			**< 51.02**				**≥ 1.157**			**< 1.157**			
**measurements**	**21**			**21**				**22**			**20**			
**percentiles**	**25%**	**median**	**75%**	**25%**	**median**	**75%**	**p**	**25%**	**median**	**75%**	**25%**	**median**	**75%**	**p**
Pentane*	11.45	15.11	20.27	11.43	15.79	20.70	0.839*	12.41	17.67	20.14	11.38	15.54	20.78	0.426*
Hexane*	†	7.99	11.14	†	†	9.25	0.221*	†	8.12	11.10	†	†	9.15	**0.07**
Heptane*	6.85	7.45	9.70	6.66	8.04	9.28	**0.94**	7.02	7.63	10.62	5.86	7.15	8.75	0.073*
Acetone*	799.26	1011.08	1305.37	291.71	383.92	501.73	**<0.001**	744.34	1007.45	1295.41	290.37	370.35	485.57	**<0.001**
**2-Butanone**	87.85	125.45	193.84	47.87	62.05	104.01	**0.007**	68.67	125.14	191.82	46.50	69.65	104.25	**0.012**
**2-Pentanone**	347.69	476.16	585.54	209.40	280.80	315.93	**<0.001**	331.36	479.08	573.15	200.64	281.89	315.15	**<0.001**
**2-Hexanone**	139.44	178.42	212.50	61.14	94.08	119.06	**<0.001**	141.33	178.82	221.09	65.81	91.67	116.33	**<0.001**
2-Heptanone*	33.08	38.79	45.64	22.55	26.04	34.42	0.005*	33.68	38.76	45.86	22.96	25.98	33.81	0.005*
3-Octanone*	47.89	56.37	85.87	36.40	56.19	82.12	0.718*	52.48	59.19	88.40	30.68	49.18	78.29	0.091*
**2-Butanone, 3-methyl-**	26.26	32.89	39.74	25.59	36.20	111.90	**0.237**	26.90	32.65	39.49	23.88	39.79	113.00	**0.399**
**2-Pentatone, 3-methyl-**	26.30	34.37	53.09	16.07	19.41	28.06	**<0.001**	28.42	35.71	62.36	15.97	18.91	25.10	**<0.001**
Methyl Isobutyl Ketone*	11.57	13.51	18.23	6.35	9.00	11.36	<0.001*	12.49	14.64	19.64	6.24	8.76	10.41	**<0.001**
Isoprene*	19.87	29.60	37.19	8.92	16.18	22.30	0.009*	24.37	33.72	37.96	6.85	13.58	20.17	**<0.001**
Acetic acid, methyl ester*	46.57	55.47	68.61	49.83	56.22	67.18	0.898*	44.16	53.06	67.61	51.26	57.65	67.51	0.494*
**Sulfide, dimethyl**	409.76	815.52	1099.63	127.23	184.20	258.03	**<0.001**	377.90	836.66	1158.19	123.61	182.72	241.42	**<0.001**
Disulfide, dimethyl*	0.37	1.12	6.44	†	0.23	0.68	**0.005**	0.41	1.21	5.72	†	0.22	0.74	**0.005**
Furan*	1.46	1.70	1.79	1.52	1.84	3.22	**0.208**	1.47	1.72	1.99	1.53	1.82	2.94	**0.332**
Furan, 2-ethyl-*	7.43	9.96	11.89	5.26	6.63	7.75	<0.001*	7.80	9.97	12.30	5.23	6.40	7.50	**<0.001**
Furan, 2-methyl-*	14.72	19.98	23.68	12.41	15.23	17.90	0.046*	15.36	19.78	24.46	12.74	15.15	17.93	0.007*
Furan, 3-methyl-*	23.41	32.32	41.93	19.33	21.37	25.66	**<0.001**	27.69	33.00	43.16	18.63	19.96	24.03	**<0.001**
Furan, 2-pentyl-*	21.99	29.25	33.91	23.11	26.73	30.05	0.454*	24.74	29.45	34.33	20.85	24.64	28.64	0.099*

p-values from Mann-Whitney-U-tests

^†^ value lower than limit of detection

* p-values from t-tests

PCA scatterplots in Fig 2b reflect the correlations of VOC profiles from feces and MAP-specific antibodies and interferon-γ response. PC1 explained 25% and PC2 15% of the variance within the data set. Loadings of PCA showed that 2-pentanone, 2-hexanone, methyl-isobutyl-ketone, 2-heptanone, 2- butanone, 2-ethyl-furan, 3-methyl-furan had the highest impact on PC1. Dimethylsulfide, dimethyldisulfide, dimethyltrisulfide, 2-methyl-furan and 3-methyl-2-pentanone had the highest impact on PC2. S/P-ratios of antibody titer and stimulation index of interferon-γ response were positively correlated to PC 1. No correlation was found between the immunological parameters and PC 2. As seen in Table 5 concentrations of acetone, 2-butanone, 2-pentanone, 2-hexanone, 2-heptanone, 3-methyl-2-pentanone, methyl isobutyl ketone, isoprene, dimethylsulfide, dimethyldisulfide, 2-ethylfuran, 2-methylfuran and 3-methylfuran were higher in animals with higher antibody titer and stimulation index of interferon-γ response.

## Discussion

VOCs from exhaled breath and headspace over feces were analyzed in an animal study in 42 goats. Twenty six animals had been inoculated with MAP 3–4 months before the first measurement and 16 non-inoculated animals served as a control group.

Distinct VOC patterns in breath and feces of infected and healthy animals could be detected by means of adapted microextraction pre-concentration techniques and GC-MS. Twenty eight VOCs were identified as potential volatile biomarkers for MAP infection *in vivo*. Differences of VOC concentrations in the headspace over feces were pronounced and reflected presence of MAP bacteria. Some of these indicative VOCs had also been found in the headspace over MAP cultures in a former *in vitro* study [9]. Differences in VOC profiles from breath were less pronounced as VOC profiles from feces and seemed to be linked to the host response in terms of interferon-γ concentrations. Although patterns of most prominent substances such as furans, oxygenated substances and hydrocarbons changed in the course of infection, differences between inoculated and non-inoculated animals remained detectable for 16 substances in feces but only 3 VOCs in breath.

Unequivocal identification and reliable quantification is mandatory in order to decide which of these volatile emissions [11, 29] can reliably indicate bacterial presence or growth. Hence, in this study, substance identification was confirmed by analysis of pure reference substances and did not solely rely on MS-database (e.g. NIST) search. Any quantification was done through calibration with reference materials.

A previous study based on differential ion-mobility spectrometry (DMS) [8] suggested differences between VOC ‘features’ in the breath and the headspace over feces from infected and non-infected animals. Due to its principal mode of action DMS is not able to unequivocally identify any substances. As numerous contaminations in order of magnitudes higher concentrations than actual marker substances such as 1-propanol may occur in the *in vivo* setting, results from unspecific methods such as DMS may easily be biased.

In general, volatile metabolites having different concentrations in feces of inoculated and not inoculated animals can be generated by bacteria or the host organism itself. Around 60% of the solid content of feces consists of bacteria [30]. Therefore, samples from feces reflect the composition and activity of the gut microbiota, which comprises the highest quantities of bacteria in the whole body. Thus, analysis of headspace over feces has the potential to provide information on characteristics of the gut microbiota. The presence and metabolism of mycobacteria which do not belong to its physiological components are likely to alter the volatile signature. As a prerequisite for identifying changes in the VOC profile caused by the presence of MAP bacteria, knowledge on commensal bacteria is necessary. In our study, we met this point by measuring a control group of animals bred and living under identical conditions as the inoculated group in a well-established animal model [7].

Concentrations of acetone, 2-butanone, 2-pentanone, 2-hexanone, 2-heptanone, 3-methyl-2-pentatone, methyl isobutyl ketone and isoprene in headspace over feces were constantly higher in the inoculated group. Potential sources of these compounds are MAP metabolism and/or host response to chronic inflammation. Aldehydes and ketones are known to be linked to oxidative or inflammatory activity on the cellular level [6]. On the other hand, mycobacteria have specific cell wall structures which account for many of the unique properties of this bacterial species. Clinically relevant characteristics include resistance to bactericidal effects or functions of the host immune system and low permeability to antibiotics. Mycolic acids represent important components of the cell envelope of mycobacteria. Derivatives of mycolic acids occur in significant amounts. Among these compounds are long chain fatty acids, alcohols and ketones [31]. In addition, aldehydes may play a role as intermediates in the biosynthesis of mycobacterial lipid metabolites [32].

Furan derivatives have already been described as metabolites from various microorganisms [29, 33–37]. Mycobacterial surface glycolipids contain D-galactofuran [38] and arabinofuranosyl residues [39]. From these pentose compounds, furfural and furans can be derived. Furans can also be synthesized from acetyl-CoA building blocks [29] or along oxidative degradation of fatty acids. I.e. furans are likely to indicate (myco-)bacterial growth as they mirror cell wall turn-over. Since substituted furans can be derived from D-galactofuran or arabinofuranosyl residues, which represent mycobacterial surface structures, these compounds may even be assigned to the growth of distinct mycobacteria. In a recent *in vitro* study we detected concentrations as high as ppmV of 2-methylfuran, 2-ethylfuran and 2-pentylfuran in MAP cultures [9]. Elevated concentrations of 2-ethylfuran, 2-methylfuran and 3-methylfuran in feces of the inoculated animals may thus be attributed to presence and growth of MAP in the gut of these animals.

3-methyl-2-butanone was the only substance having lower concentrations in the inoculated group. This substance is generated during the biosynthesis of fatty acids [29] and, therefore, represents a general marker of (bacterial) growth rather than a bacteria specific substance. Lower quantities of this marker may indicate reduced replication or decreased amounts of overall bacteria (other than MAP) in the inoculated group.

The appearance of higher dimethylsulfide concentrations in MAP-infected animals could be the result of a reduced immune defense which may have affected the composition of the gut microbiota. Ubiquitously distributed Dinoflagellates are microorganisms often associated to human illnesses [40]. Their co-existence with bacteria enables the aerobic degradation of 3-dimethylsulfoniopropionate (3DMSP) to dimethylsulfide [41–43]. 3DMSP is known to be generated from L-methionine from higher plants which, being components of hay, belong to the goat´s diet [44]. Direct catabolic degradation of L-methionine to dimethylsulfide by lactic acid bacteria is also probable [45–48].

When animals were grouped according to the immunological host response distinction between groups became more pronounced. Distinction mainly relied on the same substances which had been identified to differentiate between inoculated and non-inoculated animals. This could be due to a higher amount of MAP bacteria in those animals with higher inflammatory immune response. On the other hand, inflammatory response with generation of volatile aldehydes and ketones in the gut might as well have contributed to this effect.

In contrast to VOC patterns from feces, exhaled VOC patterns did not show pronounced differences between healthy and inoculated animals.

From the PCAs it is obvious that the actual immunological response and the age of the animals had a more pronounced effect on exhaled VOC profiles than the inoculation with MAP bacteria. Surprisingly, concentrations of differentiating VOCs were consistently higher in the group of animals with lower antibody ratios and lower interferon-γ responses. This was true for oxygenated substances related to lipid peroxidation or metabolic stress, such as aldehydes or ketones, as well as for substances typically related to environmental effects, such as benzene.

When compared to our recent *in vitro* results, 15 substances from the bacterial *in vitro* pattern could be found in the headspace of feces, but only 5 substances were detectable in the breath of the animals [9]. VOCs produced by MAP in the gut are likely to be metabolized in the liver when they enter systemic circulation via the portal vein. I.e. concentrations of reactive substances such as unsaturated hydrocarbons, aldehydes, ketones or furans potentially synthesized by MAP in the gut will be vanishingly low in breath. These results demonstrate that a direct transfer from *in vitro* biomarker to *in vivo* conditions [24, 25] is not possible and that physiology and metabolism must be taken into account.

Apart from bacterial presence and immunological response other factors including physiology, age and nutrition may influence VOC-patterns. The influence of nutrition was minimized, as the study started only after the transition from milk to plant feeding was completed and all animals received identical diet. Nevertheless the age of the animals between 21 and 55 weeks of life had a distinct effect on VOC-patterns. Although VOC profiles from feces changed markedly over time, distinction between inoculated and non-inoculated animals was still possible at every time point.

## Conclusion

VOC concentrations in the headspace over feces showed differences between infected and healthy animals. Discriminating VOCs could be linked to MAP membrane structures and metabolism. Differences in VOC profiles from breath were less pronounced and seemed to be linked to the host response. Transfer of results from *in vitro* VOC studies to *in vivo* conditions need careful consideration of related biochemistry, physiology and potential confounding parameters. Concentrations patterns of volatile marker substances determined from feces and breath changed in the course of infection. Nevertheless, differences between inoculated and non-inoculated animals remained detectable at any time. In a perspective, VOC profiles from feces may be used to recognize mycobacterial presence in the gut of ruminants.

## Supporting Information

S1 Fig Loading-plot referring to PCA-analysis of breath gas samples in relation to blood tests (Fig 2a). (TIF)

S2 Fig Loading-plot referring to PCA-analysis of feces samples in relation to blood tests (Fig 2b). (TIF)

S3 Fig Loading-plot referring to PCA-analysis of feces samples (Fig 4). (TIF)

S4 Fig Loading-plot referring to PCA-analysis of breath gas samples (Fig 5). (TIF)
