# Supplementary figures and images for: In Vivo Volatile Organic Compound Signatures of Mycobacterium avium subsp. paratuberculosis
Source: PLoS One. 2015 Apr 27;10(4):e0123980. doi: 10.1371/journal.pone.0123980 (PMC4411140; doi:10.1371/journal.pone.0123980)

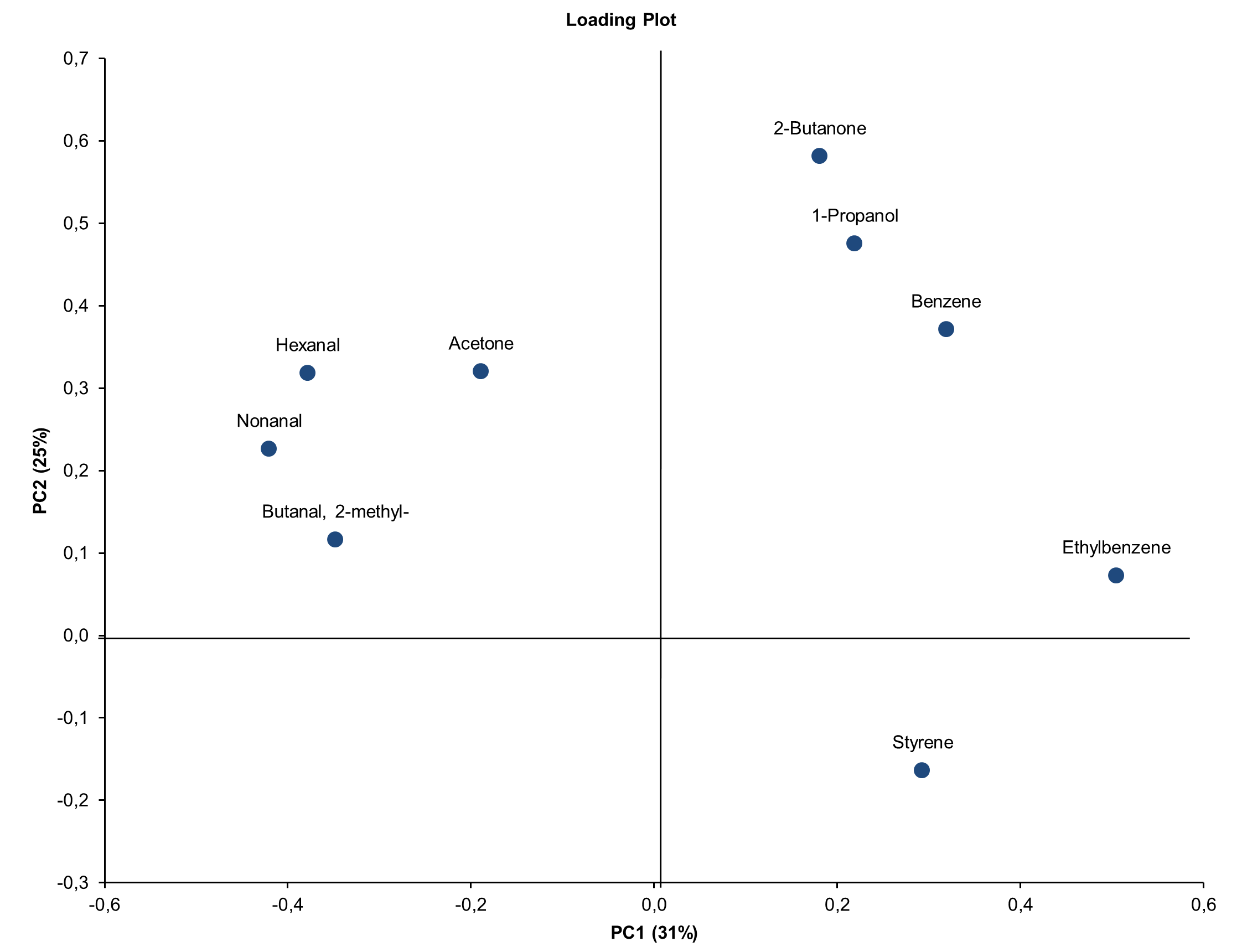

Supplement: S1 Fig — (TIF) [file pone.0123980.s001.tif]

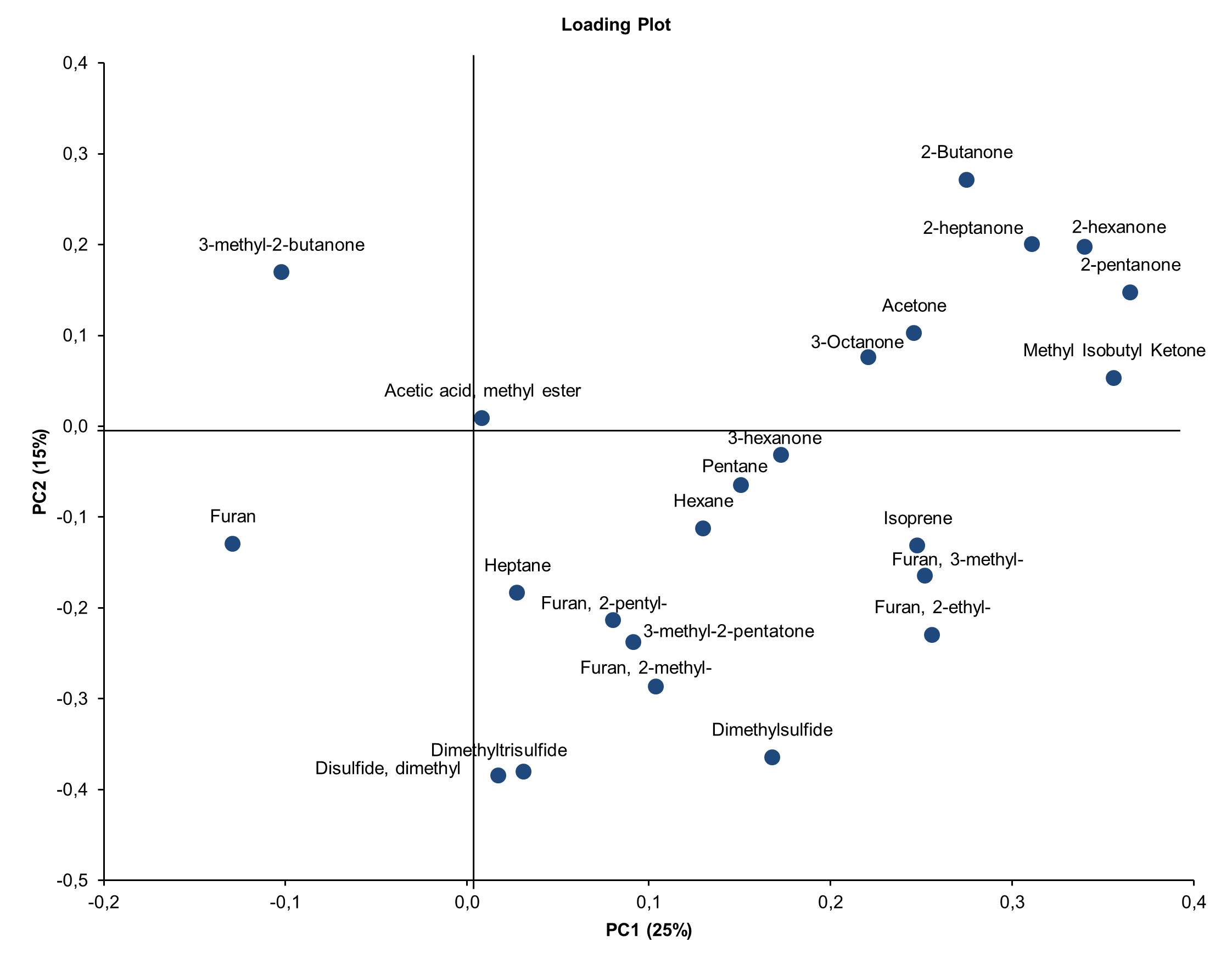

Supplement: S2 Fig — (TIF) [file pone.0123980.s002.tif]

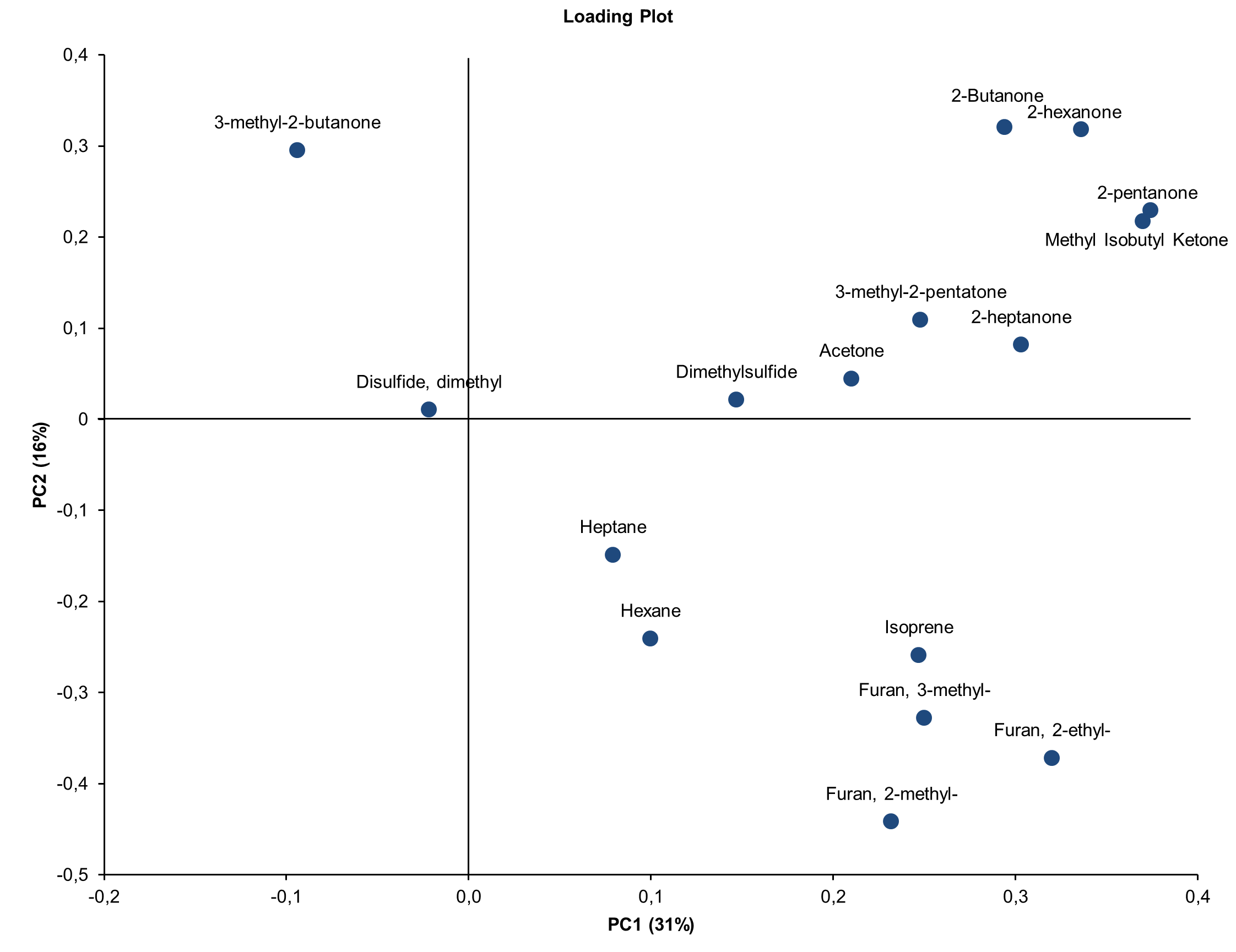

Supplement: S3 Fig — (TIF) [file pone.0123980.s003.tif]

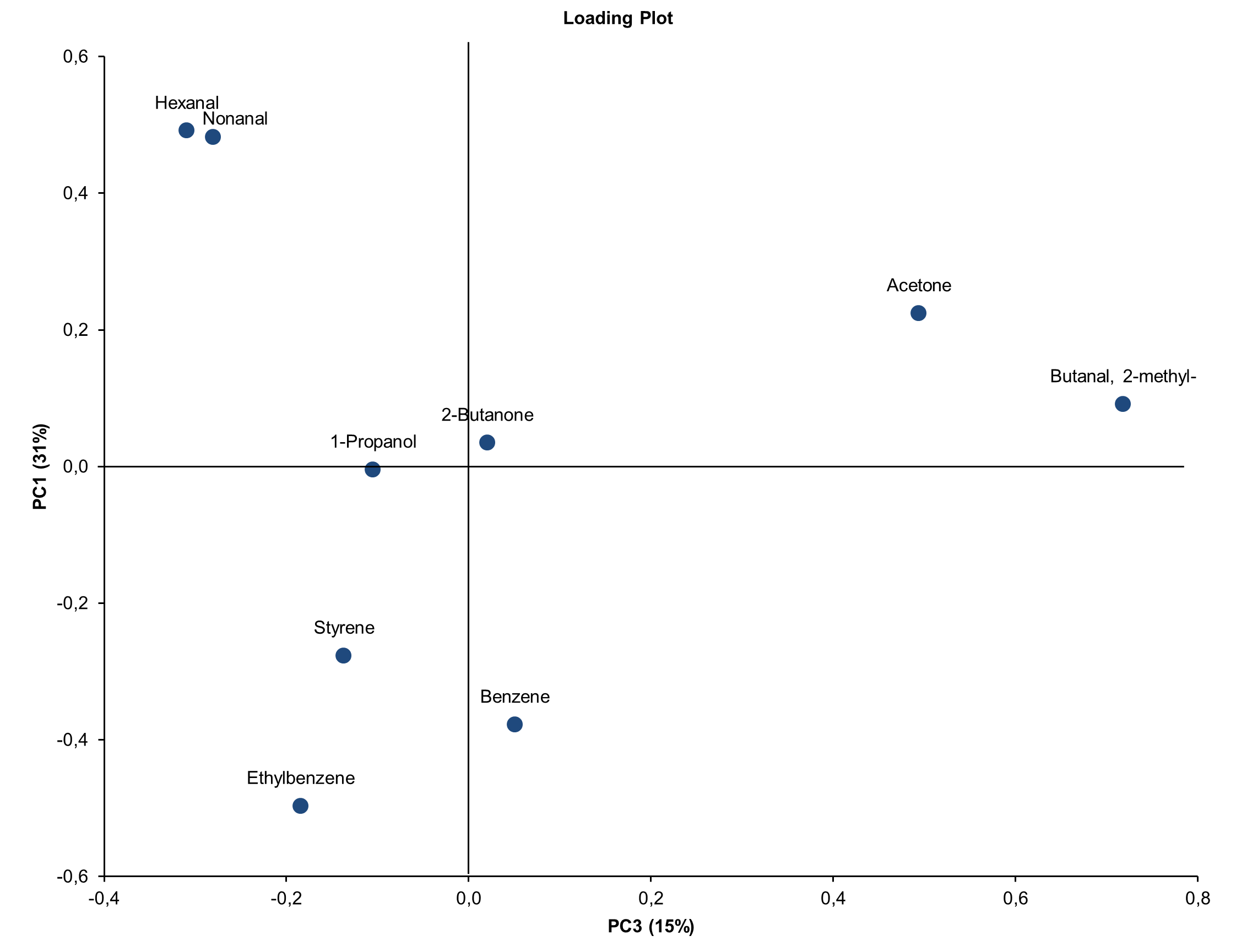

Supplement: S4 Fig — (TIF) [file pone.0123980.s004.tif]
